# Editorial: Insights in cardiovascular epidemiology and prevention: 2022

**DOI:** 10.3389/fcvm.2023.1151064

**Published:** 2023-02-10

**Authors:** Gen-Min Lin

**Affiliations:** ^1^Department of Medicine, Hualien Armed Forces General Hospital, Hualien City, Taiwan; ^2^Department of Medicine, Tri-Service General Hospital, National Defense Medical Center, Taipei, Taiwan

**Keywords:** editorial, electrocardiography, denture use, left atrial enlargement (LAE), left ventricular diastolic dysfunction (LVDD), smoker paradox, machine learning, cardiovascular diseases

This Research Topic is focused on new insights, novel developments, current challenges, latest discoveries, recent advances, and future perspectives in the field of cardiovascular epidemiology and prevention.

Four high-quality studies were included in the Research Topic. Two studies utilized electrocardiographic (ECG) variables and some biologic variables in specific models to correlate to left atrial enlargement and left ventricular diastolic dysfunction, separately, in a military sample of physically active young adults in Taiwan (1–7).

Hsu et al. used three machine learning classifiers, i.e., support vector machine (SVM), logistic regression (LR) and multilayer perceptron (MLP) for 26 ECG features with or without six biological features (age, anthropometric and hemodynamic variables) to predict echocardiographic left atrial enlargement which was defined as the dimension ≥4 cm. The comparison model used the P wave duration of lead II, the traditional ECG criterion for left atrial enlargement. The results found that if the sensitivity is fixed to 70–75%, the specificity of the SVM classifier for ECG only is 72.4%, and that of the MLP classifier for all biological and ECG features is up to 81.1%, both of which are higher than 48.8% by the P wave duration.

In another study, Liu et al. used multiple logistic regression model to identify the independent ECG and cardio-metabolic risk factors of left ventricular diastolic dysfunction, which was diagnosed as either one of the three echocardiographic criteria met: (1) mitral inflow E/A ratio <0.8 with a peak E velocity of >50 cm/s, (2) tissue Doppler lateral mitral annulus e′ <10 cm/s, and (3) E/e′ ratio >14. The results showed that of the cardio-metabolic markers, central obesity, defined as waist circumference ≥90 cm for men, was the only independent marker of left ventricular diastolic dysfunction [odds ratio (OR) and 95% confidence interval: 2.97 (1.63–5.41)]. There were no association for hypertension, prediabetes, and dyslipidemia. Of the ECG markers, left atrial enlargement and incomplete right bundle branch block/intraventricular conduction delay were the independent ECG markers of left ventricular diastolic dysfunction [OR: 2.98 (1.28–6.94) and 1.94 (1.09–3.47), respectively].

The study conducted by Limpijankit et al. aimed to investigate the association of smoking status on recurrent major adverse cardiovascular events (MACEs) in patients who recently underwent percutaneous coronary intervention (PCI). A propensity score model using inverse probability weighting with regression adjustment was used to estimate the effect of smoking on the MACE occurrence. The results revealed that MACE rates were 1.9, 1.2, and 1.6 per 100 patients per month in the current smokers, former smokers and never smokers, respectively. After applying a propensity score model, current and former smokers had the onset of recurrent MACEs sooner than non-smokers, with a median time of 4.4 vs. 4.9 vs. 13.5 months (*p* < 0.001), respectively. This study concluded that the “smoker's paradox” phenomenon (8) was not found in their population.

Finally, the study conducted by Liang et al. aimed to investigate whether denture use is associated with cardiovascular diseases (CVD) in American adults from the United States National Health and Nutrition Examination Survey (NHANE). The results revealed that denture use was associated with CVD [OR: 1.82 (1.15–2.88)] after adjustments, and the association was found stronger in women [OR: 2.13 (1.10–4.11)].

Taken together, the present Research Topic represents an important source of up-to-date information, covering many aspects of risk factors for heart diseases. More comprehensive knowledge based on these discoveries may bring about new perspectives.

## Author contributions

The author confirms being the sole contributor of this work and has approved it for publication.
